# Comparative transcriptomic analysis and endocuticular protein gene expression of alate adults, workers and soldiers of the termite *Reticulitermes aculabialis*

**DOI:** 10.1186/s12864-019-6149-4

**Published:** 2019-10-15

**Authors:** Humaira Rasheed, Chenxu Ye, Yufeng Meng, Yuehua Ran, Jing Li, Xiaohong Su

**Affiliations:** 1 Key Laboratory of Resource Biology and Biotechnology in Western China (Northwest University), Ministry of Education, Xi’an, China; 2 College of Life Sciences, Northwest University, Xi’an, China

**Keywords:** Transcriptome, Gene expression, Endocuticular protein, *Reticulitermes aculabialis*, Caste differentiation

## Abstract

**Background:**

The insect cuticle is mainly composed of exocuticle and endocuticle layers that consist of a large number of structural proteins. The thickness and synthesis of the exocuticle depend on different castes that perform various functions in alates, workers and soldiers. However, it is not clear whether the soft endocuticle is involved in the division of labour in termite colonies. To reveal the structural characteristics of the endocuticle in different castes, we investigated the thickness of endocuticle layers in alates, workers and soldiers of the termite *Reticulitermes aculabialis*, and then we sequenced their transcriptome and detected the endocuticle protein genes. The differential expression levels of the endocuticular protein genes were confirmed in the three castes.

**Results:**

We found that there was a great difference in the thickness of the endocuticle among the alates, soldiers and workers. The thickest endocuticle layers were found in the heads of the workers 7.88 ± 1.67 μm. The endocuticle layer in the head of the workers was approximately three-fold and nine-fold thicker than that in the heads of soldiers and alates, respectively. The thinnest endocuticle layers occurred in the head, thorax and abdomen of alates, which were 0.86 ± 0.15, 0.76 ± 0.24 and 0.52 ± 0.17 μm thick, respectively, and had no significant differences. A total of 43,531,650 clean sequencing reads was obtained, and 89,475 unigenes were assembled. Of the 70 identified cuticular protein genes, 10 endocuticular genes that belong to the RR-1 family were selected. qRT-PCR analysis of the five endocuticular genes (SgAbd-2, SgAbd-9, Abd-5, SgAbd-2-like and Abd-4-like) revealed that the endocuticle genes were more highly expressed in workers than in soldiers and alates.

**Conclusion:**

These results suggest that SgAbd and Abd are the key components of the endocuticle. We infer that the thicker endocuticle in workers is helpful for them to perform their functions against environmental stress.

## Background

The evolution of eusociality in insects remains one of the great mysteries of biology. Colony performance is primarily focused on the division of labour ultimately led to the placement of specific functions and behavioural professions among different castes. Termites are completely social insects, with either an exceptional frame of morphological types. Specific caste performs different tasks non-reproductives such as, workers are responsible for tending or immatures feeding and nest construction, soldier performed specific functions of defence and reproductives which allow dispersal, fecundity and pair bonding. New colonies of termites are established by the alates in the swarming season. Darkened pigmentation, a tough cuticle, and black wings categorized the alates. Soldiers typically have large, strongly sclerotic heads and with strong, highly modified mandibles. There is no doubt that the different functions of various castes are related to the significant differences in exomorphic aspects. However, we know little about how the internal structures are involved in caste development and evolution. We consider that the differences in the internal structures of individuals may be the basis of caste differentiation and the division of labour in termite colonies.

Various functional and morphological distinct layers combined to make cuticle [1]. The outer exocuticle and inner endocuticle are generally combined with those layers that were developed before and after moulting process [2]. The exoskeleton of insect cuticle is an exceptional extracellular structure which is emanate by epidermal cells and covers the outer body of insect. It serves as attachment site for muscles, implement sensory perceptions and coloration, also provide protection against parasites, pathogen, mechanical injury and perform other physiological functions. However, the cuticle mandatorily protects the whole body of insect and exist at all developmental stages, it also contains exceptional diversity in its mechanical and physicochemical properties to accommodate the functions of protected organs and tissues and enables for growth [3]. Thus, cuticle at different developmental stages from distinct parts of insect’s anatomy may have different characteristics, their functions based on the thickness, framework or morphology of unique layers, chemical composition and molecular connections in the cuticle [2, 4].

Exocuticle and endocuticle layers are composed of polysaccharide chitin and structural cuticular proteins. For example, recent studies suggested that RR-1 proteins are related to soft and flexible endocuticle and RR-2 proteins are related to tough and hard exocuticle. At different developmental stages particular cuticular protein expression are mandatory for the formation of distinct cuticle layers that may likely contains appropriate combination of morphological, mechanical and physical properties which give mobility, protection and structural support [3].

Even though a lot of data is accessible regarding the structure of the termite cuticle, studies focus on the exocuticle of various castes, because it is easy to see the difference in sclerotized and pigmented exocuticles among the alates, soldiers and workers [3, 5, 6]. Until now, the developmental pattern of the endocuticle in termite was unknown. In particular, it is not clear whether the soft endocuticle is involved in caste differentiation. *Reticulitermes* is an economically important genus and probably the most studied of all termites. For *Reticulitermes,* termitologists have established a basal developmental schema comprising two undifferentiated larval instars, followed by a bifurcated development into either (i) nymphal pathway, or (ii) the apterous pathway, which generally leads to worker termites. The nymphs follow the reproductive pathway to develop into alate adults, which are the primary reproductives that swarm to form new colonies or develop into brachypterous neotenic reproductives. The workers have unique flexibility in that a worker can develop in one of three ways: (i) into older instar workers, (ii) into sterile soldiers, or (iii) into apterous neotenic reproductives. *Reticulitermes* provides a perfect system for the study of developmental plasticity [7, 8].

In this study, to reveal the relationship between endocuticle and caste evolution, first, we compared the structural characteristics of the endocuticle of the *R. aculabialis* alates, soldiers and workers. Second, we sequenced the transcriptome of the three castes and identified the endocuticle protein genes. Finally, the differential expression level of the endocuticle protein genes via quantitative real-time reverse transcription PCR (qRT-PCR) was confirmed in alates, soldiers and workers.

## Results

### Thickness of endocuticle layers in alates, workers and soldiers

**Fig. 1:**
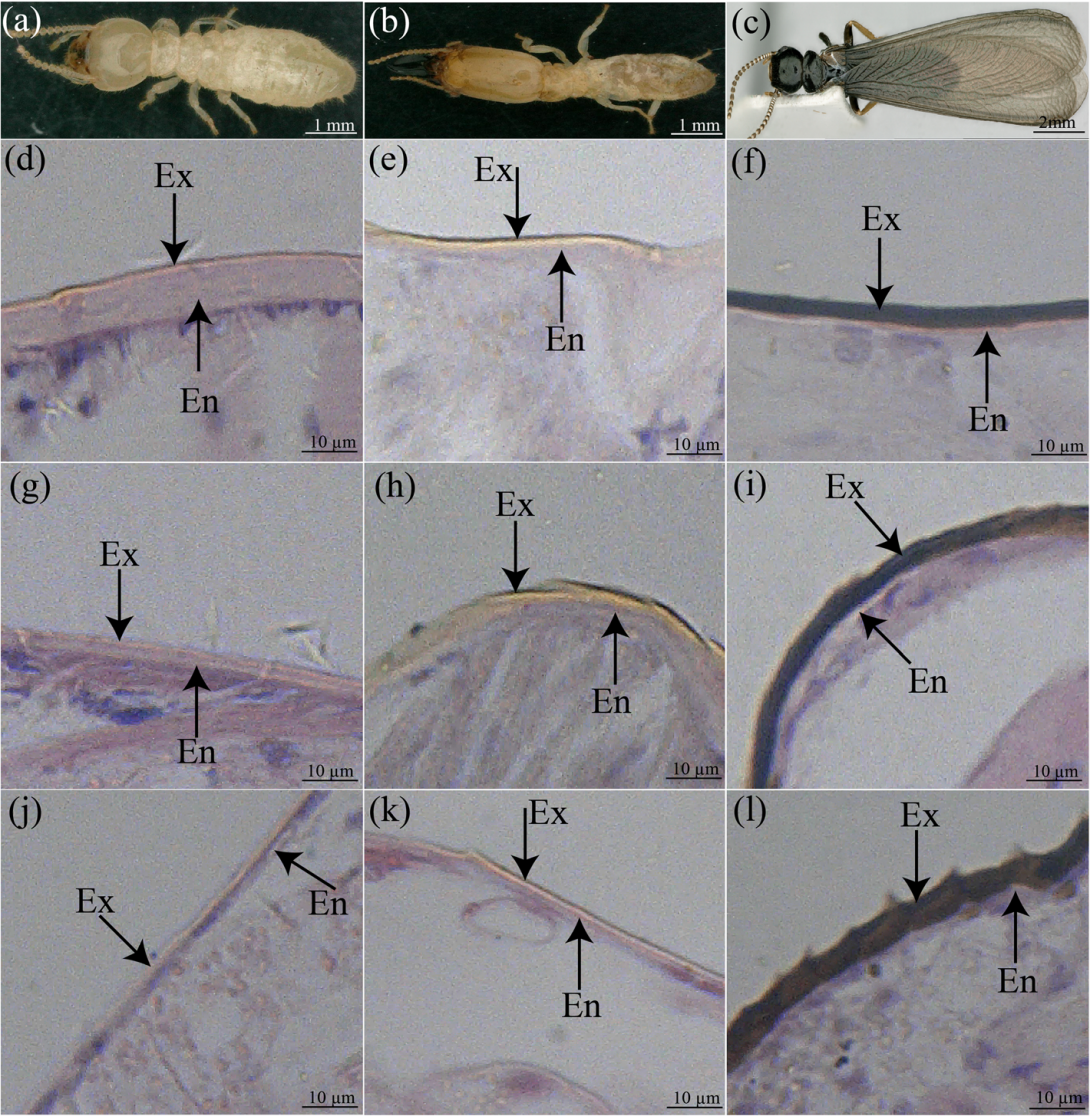
The internal morphology of cuticular layers in alates, workers and soldiers. (**a**) The worker with a white body. (**b**) The soldiers with a large dark brown head. (**c**) The alates with dark spots on body, hard cuticle and black wings. (**d**) Thickest endocuticle layer in the worker head. (**e**) Thick endocuticle layer in the soldier head (**f**) Thinnest endocuticle in the alate head. (**g**) Thin endocuticle layer in the worker thorax compared to the head (**h**) Thin endocuticle layer in the thorax of the soldier in contrast with the head (**i**) Thin endocuticle layer in the alate thorax in contrast with the head (**j**) Thin endocuticle layer in the abdomen of a worker, in contrast to their head and thorax (**k**) Thin endocuticle layer in the soldier abdomen in contrast with the head and thorax. (**l**) Thin endocuticle layer in the alate abdomen in contrast with the head and thorax

Workers were characterized by a relatively soft pale or white body. Soldiers had a light dark cuticle with dark brown coloured stripes on their heads and broad mandibles with refined solid jaws. The alates had a rigid exocuticle with black wings and natural dark black colour. Compared with the alates, the cuticles of workers and soldiers were less sclerotized and had lighter pigmentation (Fig. 1 a, b and c). The endocuticles of workers and soldiers were thicker than their exocuticles. The cuticle layer of alates contained an amazingly thick exocuticle and a very thin endocuticle (Fig. 1 d-l).

**Fig. 2:**
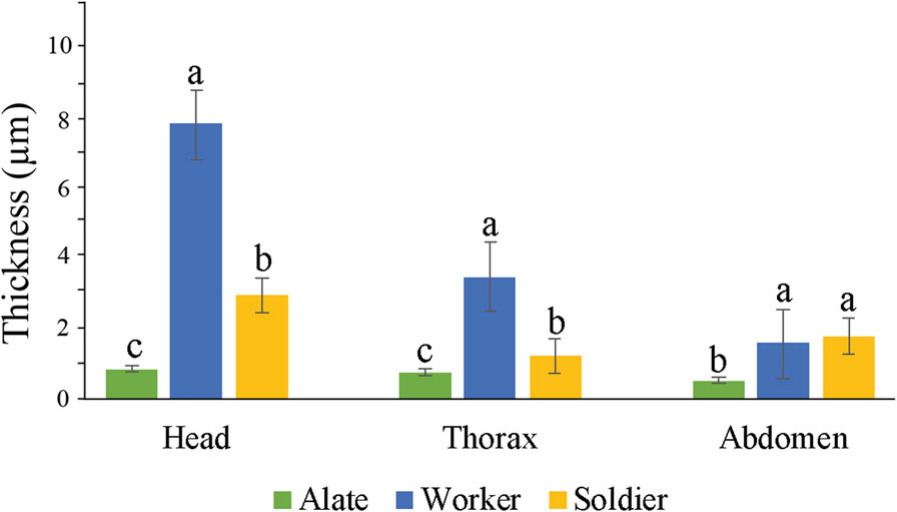
The thickness of the endocuticle layer in the head, thorax and abdomen of three castes of termites (*n* = 5). The x-axis indicates the three castes: alate, worker and soldier. The y-axis indicates the thickness of the endocuticle (mean ± SD). In the same tagmata, different alphabets on each bar indicate the significant difference between the three castes

Our data showed that in the workers, the endocuticle in heads was approximately two-fold thicker than that in thoraxes and four-fold thicker than that in abdomens. The endocuticle in the head of soldiers was two-fold thicker than in their thoraxes and abdomens, and the thickness of endocuticle in the thoraxes and abdomens of soldiers had no significant difference. Obviously, there was a great difference in the endocuticle structural characteristics among different tagmata of workers and soldiers. Interestingly, in contrast to the workers and soldiers, alates had no significant difference in the thickness of endocuticle layers among their heads, thoraxes and abdomens (Fig. 2).

There was a significant difference in the thickness of endocuticle among the alates, soldiers and workers (*n* = 5). The thinnest endocuticle layers occurred in the head, thorax and abdomen of alates, which were 0.86 ± 0.15, 0.76 ± 0.24 and 0.52 ± 0.17 μm thick, respectively, and had no significant difference. The thickest endocuticle layers were found in the heads of the workers, which was 7.88 ± 1.67 μm. The endocuticle layer in the head of the workers was approximately three-fold and nine-fold thicker than that of the heads of soldiers and alates, respectively. The endocuticle layer in the thoraxes of workers (3.5 ± 0.71 μm) was approximately three-fold and five-fold thicker than that in soldiers and alates, respectively. In spite of the fact that there was no critical contrast in the thickness of the endocuticle layer in abdomen between the worker termites (1.58 ± 0.36 μm) and soldiers (1.80 ± 0.23 μm), the endocuticle layers in the abdomens of the workers and soldiers were approximately three-fold thicker than those in alates (0.52 ± 0.17 μm) (Fig. 2).

### Illumina sequence data and assembly

We used Hi-SeqTM 2000 (Illumina) paired-end sequencing to sequence the three different castes (alate, worker and soldier) of *R. aculabialis* and acquired a total of 43,531,650 clean-sequence reads. RNA of each sample was extracted from the heads and thoraxes of 20 individuals. Each biological replicate consisted of pooled heads and thoraxes from 20 individuals, three biological replicates were used. A total of 89,475 unigenes varying from 201 to 44,333 bp were gathered by using the Trinity program (Additional file 1), which was based on clean reads. The N50 length was 1319 bp and the average length was 831 bp. For more than 98.32% of the cycles, average quality values ≥20 were procured. Approximately 45.36% GC contents of the sample were constant. According to the sequencing output results, quality was enough, and we could use this for an additional analysis.

### Transcriptome annotation of *R. aculabialis*

**Fig. 3:**
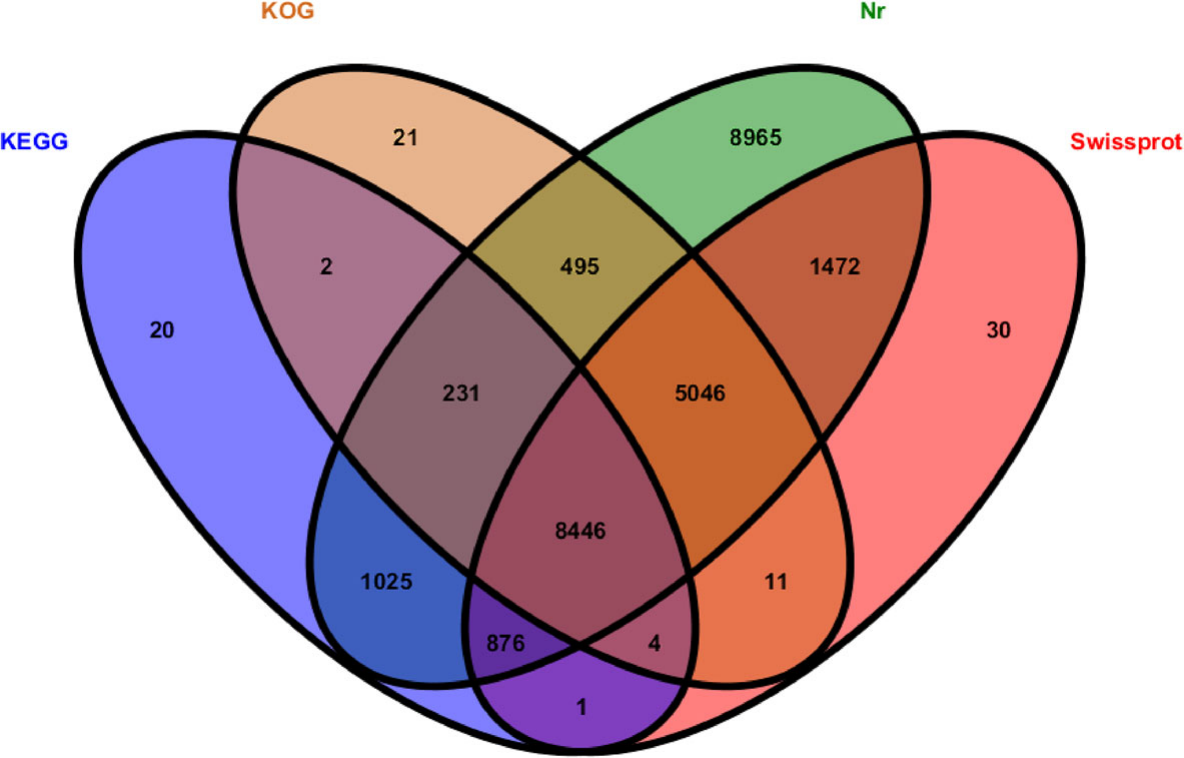
Venn diagram distributed the similarity search results, from the Nr, Swiss-Prot, KEGG and KOG databases. The overlapping quarters of the four circles showed the number of unigenes that shared BLASTx similarities in the respective databases

We annotated the sequence of 89,475 unigenes. According to the Venn diagram, the overlapping of four circles showed the unigenes number that shared the BLAST x similarities on sequence based obtained from the Nr, Swiss-Prot, KEGG and KOG databases (Fig. 3). In the Nr database, 26,556 (29.67%) unigenes of *R. aculabialis* had expressive matches; in the Swiss-Prot database, 15,886 (17.75%) unigenes had expressive matches; in the KOG database, 14,256 (15.93%) unigenes had expressive matches and 10,605 (11.85) % had expressive matches in the KEGG database (Additional file 2).

The majority of 89,475 unigene sequences could not be matched to known genes (70.22%) due to the comparatively small lengths of the discrete gene sequence and absence of genome information for *R. aculabialis*. It was also indicated from the matches to the Nr database that a large number of *R. aculabialis* unigenes closely matched the sequences of *Zootermopsis nevadensis*, *Coptotermes formosanus*, *Blattella germanica* and *Anoplophora glabripennis* (46.17, 2.55, 2.51 and 2.44%, respectively).

### Classification of the Kyoto encyclopedia of genes and genomes (KEGG), eukaryotic orthologues groups (KOG) and gene ontology (GO)

**Fig. 4:**
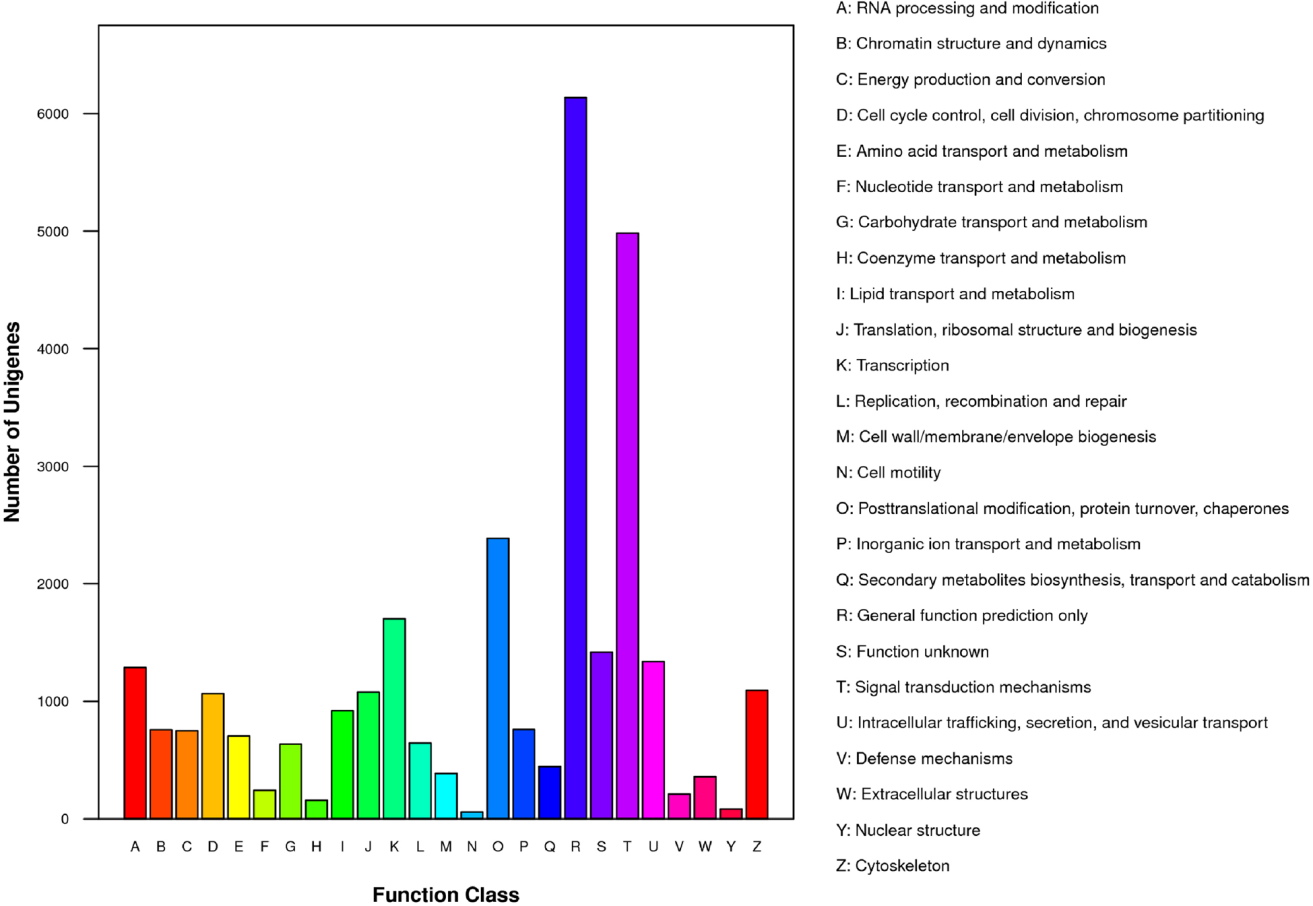
Histogram representation of eukaryotic orthologous groups classification. In 25 categories of KOG, 14,256 unigenes were annotated. The 25 groups are represented in the caption, and y-axis represents the number of unigenes in a specific functional clump

The functions of predicted unigenes were classified by using GO, KEGG and KOG analysis, which depend upon the results of protein annotation as result of the Nr database homology. A total of 25 categories of KOG were classified, in which 14,256 unigenes were annotated (Fig. 4). The largest group from the 25 categories enclosed was a general functional prediction only (6137 genes, 43.04%) followed by signal transduction mechanisms (4982 genes, 34.94%), posttranslational modification, protein turnover and chaperones (2386, 16.73%), function in transcription (1701, 11.93%), function unknown (1419, 9.95%), intracellular trafficking, secretion and vesicular transport (1340, 9.39%), RNA processing and modification (1288, 9.03%), cytoskeleton (1093, 7.66%), translation, ribosomal structure and biogenesis (1078, 7.56%), cell cycle control, cell division, chromosome partitioning (1065, 7.47%) and lipid transport metabolism (920, 6.45%) had unigenes with percentages. The smaller groups that were found in KOG were coenzyme transport and metabolism, nuclear structure and cell motility unigenes with the following percentages (157, 1.10%, 83, 0.58% and 57, 0.39%, respectively).

**Fig. 5:**
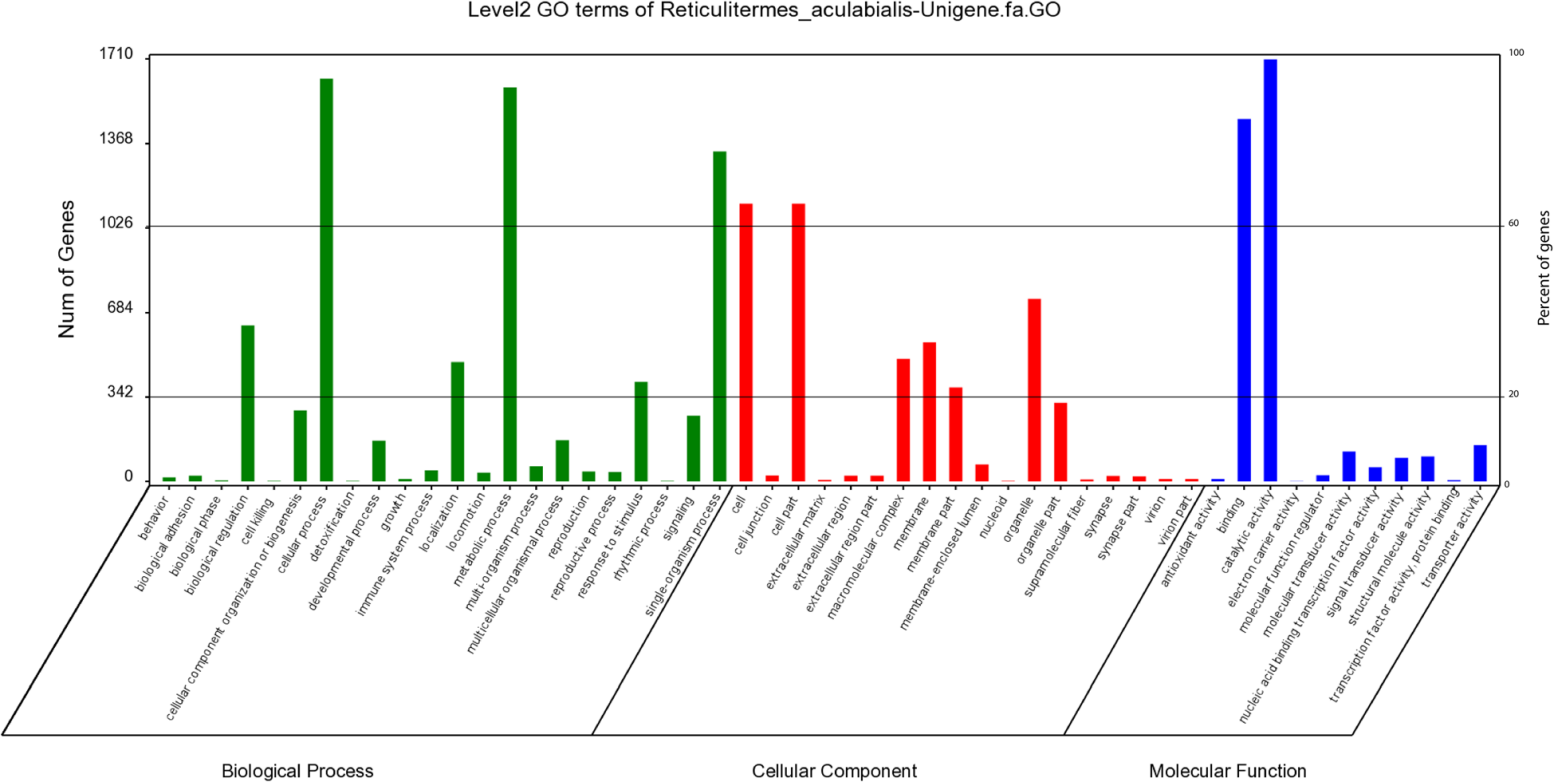
Histogram representation of Gene Ontology classification. The main groups of gene ontology biological processes, molecular functions and cellular components are describing in the histogram. The number of genes in each group is shown in the y-axis on the left side, and the percentage of a specific group of genes in the main group are shown in the y-axis on the right side

The annotated unigenes that belonged to molecular function, cellular components and biological process clusters were divided into 51 groups, which contained cellular processes, cell parts and organelle, metabolic processes, binding and catalytic activity (Fig. 5). Gene ontology annotations were categorized into three main groups i.e. cellular components, molecular functions and biological processes were obtained as 4966 (31.14%), 3738 (23.44%) and 7243 (45.41%), respectively.

**Fig. 6:**
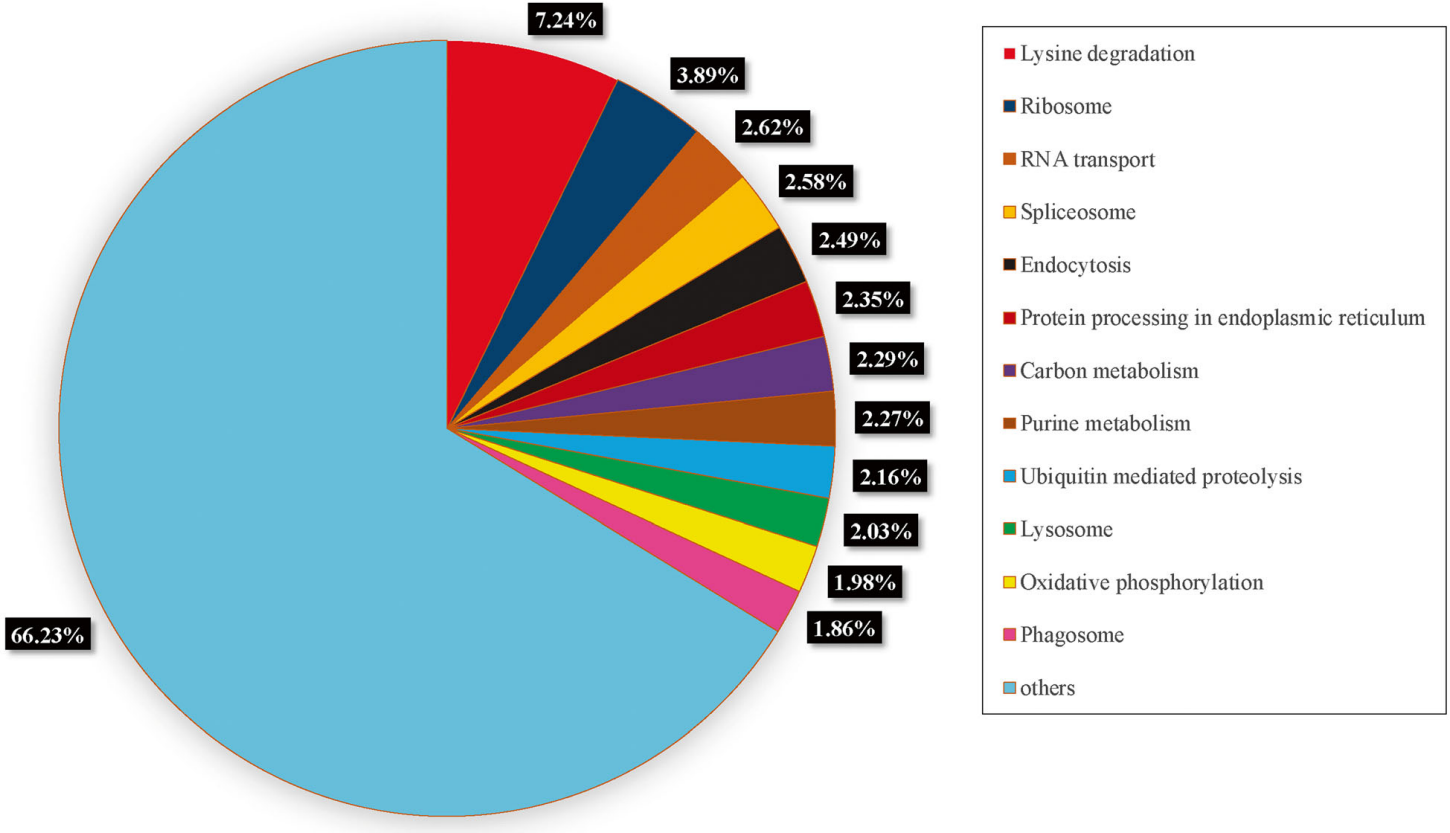
Classification of Kyoto Encyclopedia of Genes and Genomes. In 208 pathways of KEGG, 5179 unigenes were allocated. More than 150 unigenes were plotted in these pathways

To understand the biological process pathways, present in *R. aculabialis* we plotted the KEGG as a reference-authorized pathway of the unigenes sequence. A total of 208 KEGG pathways contained 5179 unigenes. Lysine degradation (610 members), ribosomes (328 members), RNA transport (221 members) and spliceosome (217 members) were the pathways that showed unique or specific sequences (Fig. 6). For exploring the structures, specific processes, functions and pathways that are included in reproductive differentiation, these annotations offer vital support.

### Differentially expressed gene (DEG) analysis in alates, workers and soldiers

**Fig. 7:**
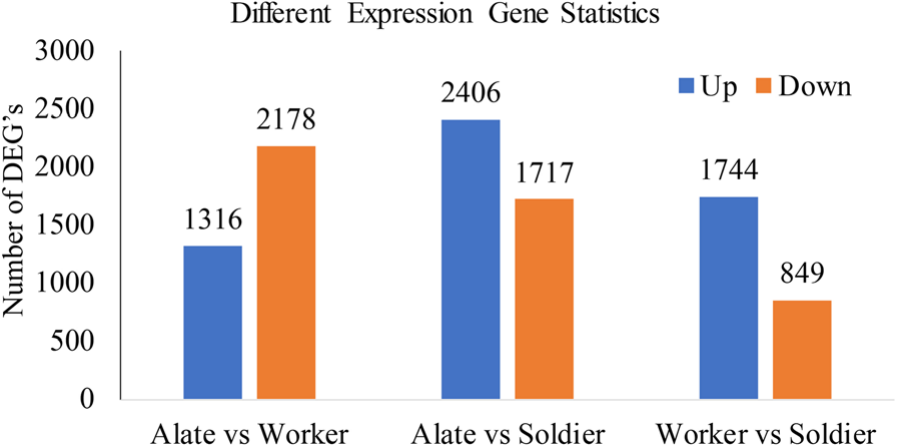
The number of differentially expressed genes in the worker, soldier and alate. Three castes (alates, workers and soldiers) are represented in the x-axis. The blue and orange transcripts were significantly upregulated and downregulated, respectively. To find the benefits of gene expression differences, two parameters log2Ratio > 1 and FDR < 0.05 were used

After the expression level of each gene was calculated (three biological replicates), differential expression analysis was conducted using edgeR. To recognize downregulated and upregulated genes between the alates, workers and soldiers we used FDR < 0.05 and the absolute value of log2Ratio ≥1 for a filtering approach. After a relative analysis between worker, soldier and alates, a total of 10,210 differentially expressed genes were separated, in which 5466 and 4744 genes were differentially upregulated and downregulated, respectively. The minimum number of differentially expressed genes were shown in “worker vs soldier” compared to the “worker vs alate” and “soldier vs alate” (Fig. 7). We used the KEGG pathway analysis and GO classification to identify the biological functions of differentially expressed genes among workers, soldiers and alates. By using BLAST2GO on annotated transcripts, a Gene Ontology analysis was completed. As a result, a total of 10,210 differentially expressed genes were divided into 51 functional categories, which were annotated in the Gene Ontology database. These 51 groups were further divided into three main categories, such as molecular functions, biological processes and cellular components. KEGG pathway analysis showed that one pathway (starch and sucrose metabolism) was significantly changed in the alates compared with soldiers; 25 pathways were significantly changed in the alates compared with workers, including ribosome, glycine, serine and threonine metabolism, phagosome, citrate cycle, MAPK signaling pathway,estrogen signaling pathway, etc.; 19 pathways were significantly changed in the workers compared with soldiers, including ribosome, phagosome, focal adhesion, regulation of actin cytoskeleton, adherens junction, etc. (Q-value < 0.05) (Additional files 3).

### Prognosis of protein coding regions (CDS)

To further analyse the functions of the unigenes at the protein level, we predicted the CDS of all of the unigenes. By using EST scan (version 3.0.2) and BLAST x (E-value < 0.00001), we predicted 2514 and 26,419 unigenes, respectively. The length distribution of the protein-coding regions prognosis from BLAST x and EST scan results were displayed in Additional files 4 and 5, respectively. The number of protein-coding regions slowly decreased as the sequence length increased, and in the unigenes assembly, it remained constant.

### Endocuticle protein genes of *R. aculabialis*

**Fig. 8:**
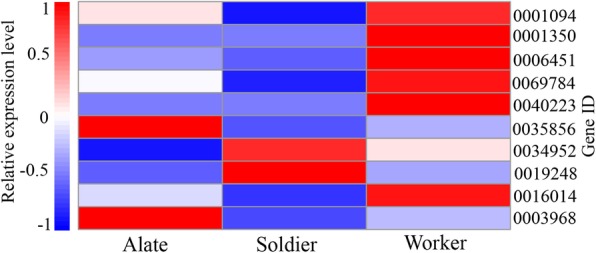
Heat map of RNA-seq transcriptome analysis for 10 endocuticle protein genes in three castes (alates, workers and soldiers) are represented in the x-axis. Three SgAbd-2 genes (Unigene 0001350, 0006451 and 0034952), two SgAbd-9 genes (Unigene 0001094 and 0016014), SgAbd-1-like gene (Unigene 0040223), two SgAbd-2-like genes (Unigene 0069784 and 0019248), Abd-5 gene (Unigene 0003968) and Abd-4-like gene (Unigene 0035856). The gene ID is represented in the y-axis right side, and the colour from red to blue indicates a high to low expression level, which is represented on the left side

Our analysis of the RNA-seq data identified 50 cuticular protein genes and 20 predicted cuticular protein genes. Of the 70 cuticular protein genes, 44 genes were similar to the cuticular protein genes of termite *Zootermopsis nevadensis.* 10 genes were annotated as endocuticle protein genes including 6 identified and 4 predicted endocuticle protein genes. Those endocuticle proteins were endocuticle structural glycoproteins including SgAbd-2, SgAbd-9, Abd-5, SgAbd-2-like, SgAbd-1-like and Abd-4-like, which were classified into the RR-1 protein group of CPR family (Additional file 6). Previous studies have suggested that RR-1 proteins are associated with soft (flexible) endocuticle [9]. Therefore, in the reproductive or non-reproductive castes of *R. aculabialis*, the 6 RR-1 proteins were related to the formation of endocuticle. Based on RNA-seq, we compared the expression profiles of ten endocuticle protein genes in the alates, workers and soldiers. The six endocuticle protein genes were expressed at higher levels in the workers than in the alates and soldiers (Fig. 8).

### qRT-PCR verification of the endocuticle protein gene expression in the alates, workers and soldiers

**Fig. 9:**
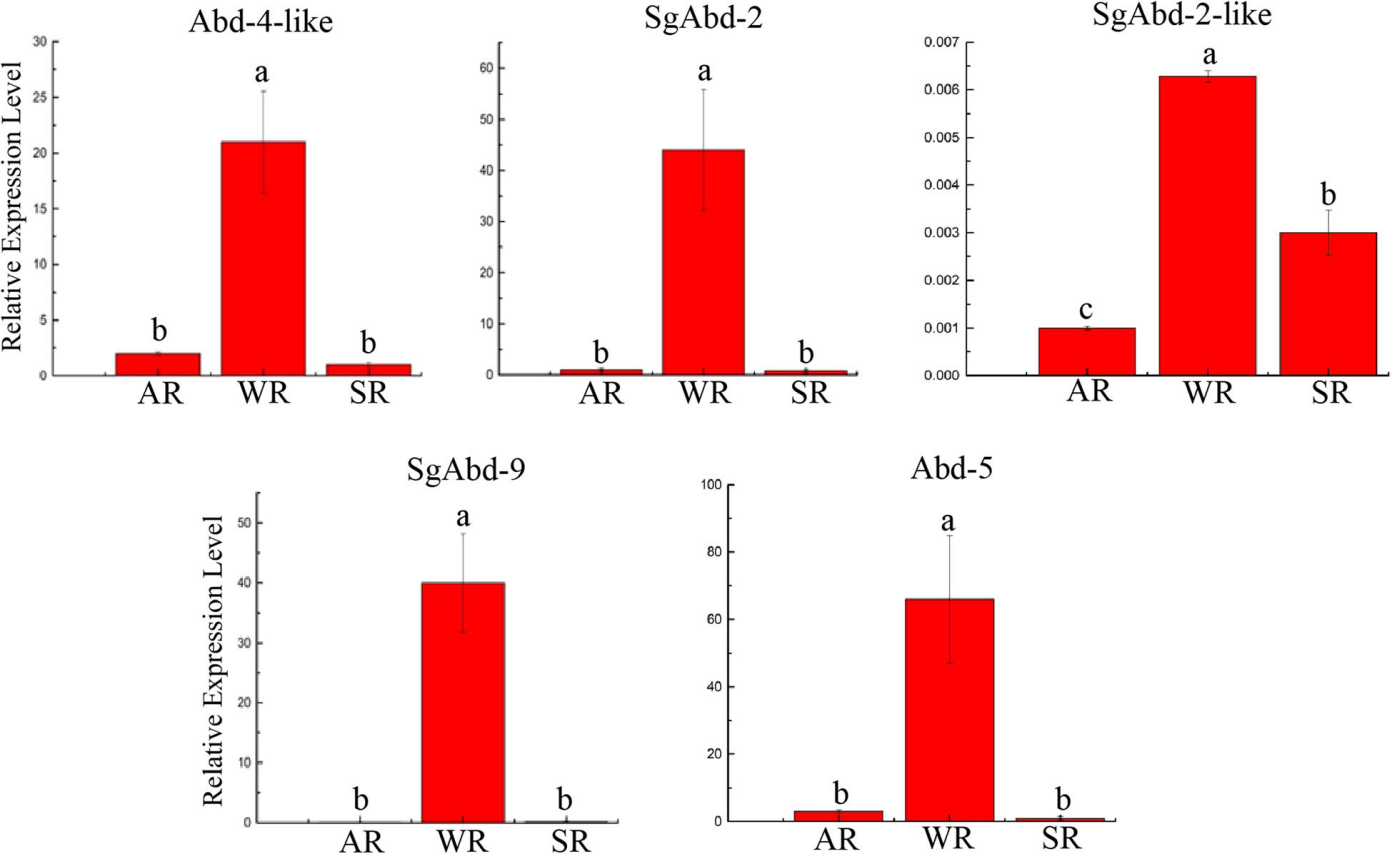
qRT-PCR analysis of the five selected genes that are involved in the endocuticle layer of three castes of *R*. *aculabialis* (AR: alates, WR: Workers and SR: Soldiers) are represented in the x-axis. Different letters, a and b, above each bar showed significantly different groups. *P*-values < 0.05 were considered significant and P-values > 0.05 were non-significant. The non-parametric Kruskal-Wallis test followed by Dunn’s multiple comparison tests were used to identify the significant differences among the groups

qRT-PCR analyses were performed for five endocuticle genes in the alates, workers and soldiers (three biological and three technical replicates). Each of the biological replicates for qPCR consisted of pooled heads and thoraxes from 20 individuals (Additional file 7). Our results suggested that the endocuticle protein genes displayed different expression patterns among various castes: (i) All endocuticle protein genes were highly expressed in workers, and (ii) Except that the expression level of the SgAbd-2-like gene in soldiers was significantly higher than in alates, there was no significant difference in the expression level of all others between the soldiers and alates (Fig. 9).

We demonstrated that five endocuticle genes (SgAbd-2, SgAbd-9, Abd-5, SgAbd-2-like and Abd-4-like) were more highly expressed in the workers than in the soldiers and alates. The expression level of SgAbd-2 in the workers was approximately 400-fold and 200-fold higher than in the alates and soldiers, respectively, and significant difference was present in the expression level of SgAbd-2 between the soldiers and alates (*P* < 0.05). The expression level of SgAbd-2-like was significantly higher in the workers than that in the soldiers and alates. The expression level of Abd-4-like in the workers was 22-fold and 66-fold higher than in the alates and soldiers, respectively. The expression level of SgAbd-9 was approximately 50-fold higher in the workers than that in the alates and soldiers, and there was no significant difference in the expression level of SgAbd-9 between the alates and soldiers. Abd-5 showed higher expression in the workers than that in the alates and soldiers, and there was no significant difference in the expression levels between the alates and soldiers (*P* > 0.05).

## Discussion

In the present study, the differences in gene expression among workers, soldiers and alates were revealed using de novo sequencing of transcriptomes. All colony members share the same genetic background, and differences in the castes are caused by differences in gene expression [3]. We confirmed a total of 10,210 differentially expressed genes among workers, soldiers and alates of *R. aculabialis*. We found that the minimum number of differentially expressed genes were shown in “worker vs soldier”. This result is consistent with their developmental characteristics in which soldiers (non-reproductive caste) are derived from workers (non-reproductive caste) and alates (reproductive caste) are derived from nymphs. According to the KEGG pathway analysis, there were significant differences in 45 pathways among the workers, soldiers and alates indicating that these pathways were related to the physiological functions of different castes.

We found that the endocuticles of workers were significantly thicker compared with those of soldiers and alates. Four layers (epicuticle, exocuticle, endocuticle and epidermis) gradually deposited on each other to form cuticle thickness [10]. A major portion of cuticular proteins in insects is enclosed in the exocuticle and endocuticle, which provides muscular support and act as a protective shield [11]. Several layers of proteins and fibrous chitins which crossing each other in a sandwich motif are combined to form a soft and flexible endocuticle [12]. The expression of specific cuticular proteins is probably required for the formation of diverse cuticles at different developmental stages that exhibit appropriate combinations of physical and morphological properties to provide structural support, mechanical protection and mobility [4, 13]. If larvae and nymphs have thin endocuticles, we can sure that the workers have thick endocuticles as an adaptation to enable them to work. If larvae and nymphs have thick endocuticles like workers, the thick endocuticle in workers is just the default. In termite colonies, the workers, alates and soldiers have distinct features in morphology, function and adaptability, which are crucial to the survival of colonies. Compared with the alates and soldiers, the cuticle of workers was less sclerotized and had lighter pigmentation. Although the exoskeleton helps to protect the insect from environmental stresses, such as predators, parasites, abrasions, desiccation, and UV radiation [14, 15], the workers with the thin exocuticle layer and thick endocuticle layer can move flexibly in the nest. Interestingly, we found that the soldiers had such a markedly reduced endocuticle in both heads and thoraxes compared with workers. Soldiers are derived from workers. Workers can develop into soldiers via two moults. Therefore, we suggest that the thickness of endocuticle in heads and thoraxes can change with the transformation of caste and the structural characteristics of endocuticle in heads and thoraxes are related to the functions of termite castes.

The alates have the thinnest endocuticle and the thickest exocuticle, which increase the hardness of the body wall, which facilitates joint movement of the muscle and pulls up the wings to fly [14, 15]. Muscles are connected to the body wall along with attachment fibres regulating throughout the epicuticle and cuticle. The movement of different parts of body, appendages such as wings depends on these muscle attachment [12, 16]. Therefore, we suggest that alates have the thinnest endocuticle layer to reduce weight for flight.

The thickest endocuticle layer was found in the heads of workers. The head of the termite is an important part of the body with thick endocuticle and thin exocuticle with highly sclerotized and thick exoskeletal head capsule to protect the brain. The head capsule of insects contains bulk of vital sensory organs with antennae and ocelli. Hairs (setae) on the cuticle are sensitive to touch, sound and vibration and controls the defence system [17]. In a previous study of *Locusta migratoria*, the whole-body transcriptome exhibits the RR-1 protein genes expression was very high in their head, and they determined that expression of these genes is continuously involved in the synthesis of cuticle [18]. We suggested that the head endocuticle of workers is thick because the head is the centre of sensory nerves, and the endocuticle is also related to sensations that involve some sensory factors. The mouthpart and head of workers are hardened, but the body is not. The workers use their mouth to dig tunnels, and the older workers are responsible for defending the colony from invaders. The thick endocuticle layers can bear pressure and protect their heads.

In this study, we demonstrated six structural endocuticular proteins (SgAbd-1-like, SgAbd-2, SgAbd-9, Abd-5, SgAbd-2-like and Abd-4-like) belonging to family RR-1, which were important in the synthesis of the endocuticle and its thickness. Based on RNA-seq, we found that most of endocuticle protein genes were expressed at higher levels in the workers than in the alates and soldiers (Fig. 8). Major portion of insect cuticle are structural cuticular proteins that perform major role in the cuticles and exoskeletons synthesis. Previously it was suggested that caste differentiation and identification of insects can be determined by cuticular proteins that were involve in maintaining the body shape and outer cuticle [4, 19]. The cuticular protein genes expression is a way to determining the thickness of cuticle [20]. For example, *BmorCPH24* in *Bombyx mori* larvae perform a key role in structural cuticular protein that helps in synthesis of larval endocuticle, and obstruction of *Bmor*CPH24 restrict the endocuticle amalgamation [21]. These endocuticular proteins are helpful by protecting *R. aculabialis* workers against environmental stress and maintenance of the body shape.

Our results showed that all of the functional genes are highly expressed in workers of *R*. *aculabialis*. This might be the major contributor in the thickness of the cuticle. Structural glycoproteins are insoluble macromolecular glycoproteins in the extracellular matrix. Insect cuticular proteins are diverse and present among all body parts and developmental stages and manifest diverse physical properties with a variety of strength and elasticity [22]. In *Tribolium castaneum* the RNA interference results showed that the endocuticle protein gene *TcCPR4/27* was remarkably thick in the young elytra as compared to adult elytra [23]. The same results were found in previous studies of *Bombus terrestris*, in that the expression level of the endocuticle structural glycoprotein gene in larvae was higher compared to that in adults (queen and worker stages) [24]. Therefore, we suggest that SgAbd and Abd (RR-1 proteins) are the key components for flexible, soft and hydrated endocuticle.

Termite morphological and anatomical adaptations are caste-specific, with structures evolving independently in alates (to allow dispersal, pair bonding and fecundity), workers (foraging, feeding, tending, nest construction) and soldiers (defence) [25]. Termite cuticular armament is thin and less sclerotized than that of other social insects, which renders termites more vulnerable to parasites that invade through the integument. Only the alates have well-sclerotized and pigmented cuticles, and a pair of alate adults can found a new colony without the help of workers. The cuticular protein characteristics might determine whether a reproductive individual can or cannot become a primary reproductive [3]. In this study, the thickest endocuticle layers were found in the workers and the endocuticle genes were more highly expressed in workers than in soldiers and alates. We speculate that endocuticle characteristics are related to the division of labor and environmental adaptation of termites, which is involved in the evolutionary and ecological success of termites.

Caste differentiation in termites analysed on molecular basis was significantly upgraded by the advancement in integrative, genomic and molecular biology was great achievement by scientists [25]. More experiments are needed to reveal the detailed information about molecular components, their functions and conduction of material throughout the cuticle, and how they interact with one another to shape the overall architecture and properties of the endocuticle layer will be the focus of future studies.

## Conclusion

We reported the structural characteristics and thickness of the endocuticle layers in different castes of *R. aculabialis* and sequenced the transcriptome to compare the expression levels of the endocuticle protein genes of workers, soldiers and alates. We demonstrated six structural endocuticular proteins (SgAbd-1-like, SgAbd-2, SgAbd-9, Abd-5, SgAbd-2-like and Abd-4-like) belonging to family RR-1, which were important in the synthesis of the endocuticle and were helpful for termites to perform their functions against environmental stress.

## Methods

We collected the workers, soldiers and alates of *R. aculabialis* from Northwest University, Xi’an, China, in May 2017. The heads and thoraxes of alates, workers and soldiers were cut and quickly stored in liquid nitrogen until RNA extraction.

### Observation of endocuticle

The fixed workers, soldiers and alates were dehydrated in a series of graded ethanol and then transferred into xylene. Each sample was embedded into paraffin, and then 7 μm sections were cut by using a LEICA CM1850 microtome. Poly-lysine-coated slides were used to collect the longitudinal sections. Deparaffinized and dehydrated sections were stained with haematoxylin solutions and eosin solution, and then were observed by using a VHX digital microscope. BZ-II analysis was used with application software (Keyence) to measure the thicknesses of the endocuticular layer of the tissue (in head, thorax and abdomen). Five replicates were used to obtain the average value of each individual (*n* = 5). The significant differences were identified with the non-parametric Kruskal-Wallis test followed by Dunn’s multiple comparisons test. *P*-values < 0.05 were considered as significant.

### RNA extraction and construction of cDNA library

By using RNA iso plus reagent (Takara Bio. Inc., Japan) the total RNA of the heads and thoraxes of 20 individuals of workers, soldiers and alates were extracted according to a given manufacturer method, respectively (three biological replications). After extraction, an A2100 Bio-analyser (Agilent Technologies, Santa Clara, USA) was used to measure the quality of RNA. The RNA quality (1.8–2) was used to build a cDNA library. The oligo (dT) magnetic beads (Qiagen Co., Ltd., Shanghai) were used to extract polyA mRNAs for cDNA library construction. For first- and second-strand cDNA, random hexamer-primers and buffers, dNTPs, RNase H and DNA polymerase I were used, followed by cDNA synthesis, and then the mRNAs were shattered into small fragments. The cDNA fragments were attached to the sequencing adaptors after their refinement. The cDNA was checked by using agarose gel electrophoresis, with fragment length of 200 bp. After refinement, PCR was used to build a final cDNA library.

### Sequencing and transcriptome assembly

An Illumina sequencing platform (Illumina Hi Seq TM 2500) technology (Guangzhou, China) was used to sequence the previously built cDNA. A practical extraction and report language (Perl) software was used to filter the clean reads and adaptor sequences read. Transcriptome De-novo assembly was constructed by using Trinity software (version 2.0.6) [26]. Trinity is a modular method or software package that is a combination of three constituents, such as Inchworm, Chrysalis and Butterfly used in order to expresses and differentiates full-length splicing isoforms from a pair of genes that are derived from the transcript in a single graph.

### Mapping of reads and gene expression quantification

For short sequence mapping, SOAPaligner/soap2 was used to intend the alignment the reference sequence, which is affiliated with SOAP (short oligonucleotide analysis package) and its upgraded version [27]. The expression level of a single gene was computed by the total number of reads that were covered by that gene, and the expression quantity of all exposed genes was computed by the same method as above. The reads per kilobase per million mapped reads (RPKM) tell the exact difference between the gene length and the sample sequencing. RPKM was calculated by using the following formula:

RPKM = number of reads/ (gene length/1000 × total number of reads/1,000,000).

R package was used for envisioning and expression data statistics (http://www.r-project.org/).

### Differentially expressed genes analysis and false discovery rate (FDR)

Differential expression analysis was carried out by using edgeR, which is a statistical method, applied for the differential expression of genes after measurement of the quantification of each gene as described by Chen et al. [28]. In our analysis, a minimum limit for the *p*-value was calculated by the false discovery rate. To examine the benefits of gene expression differences in the analysis, the definite values of log2Ratio > 1 and FDR < 0.05 were used.

### Gene ontology (GO) and KEGG enrichment analysis

According to Chen, previously described differentially expressed genes methods were used for Gene Ontology and KEGG enrichment analysis [29]. Unigenes were annotated by using Nr (non-redundant protein) annotation results. Cellular components, molecular function and biological processes were the three main groups of gene ontology, which contained 51 functional categories. In differentially expressed genes, the KEGG and Gene ontology pathways with Q-values < 0.05 were remarkably improved. Blast2GO software was used to analyse the annotated unigenes of gene ontology [30]. WEGO software was used for functional classification of unigenes [31].

### Quantitative real time PCR

RNA was extracted of three different castes of termite (workers, soldiers and alates), their heads and thoraxes were used to obtain total RNA by using the RNA isoplus reagent (Takara Bio. Inc., Japan). Each of the biological replicates for qPCR consisted of pooled heads and thoraxes from 20 individuals,. Quantitative real-time PCR (qRT-PCR) was used to amplified complementary DNA by using 50 ng of mRNA and prime script RTase (Takara Bio. Inc., Japan). SYBR Premix Ex TaqTM II (Takara. Bio. Inc., Japan) was used in quantitative reaction on a Light Cycler 480 software 1.2.0.0625 (Roche Diagnostic, Switzerland). Beta-actin gene was used as a reference for expression results and Ct values were used to maintain all reactions in qRT-PCR [3, 32]. The 2^−∆∆Ct^ method was used to measure the corresponding gene expressions [3, 32]. We used the non-parametric Kruskal-Wallis test combined with post hoc Dunn’s multiple to compare the significant differences among three castes of *R. aculabialis* with three biological and three technical replicates in all qRT-PCR experiments.

## Supplementary information

**Additional file 1. **Unigenes length distribution of the *R. aculabialis* unigenes.

**Additional file 2. **Functional annotation of the *R. aculabialis* transcriptome.

**Additional file 3.** DEGs enrichment by the KEGG pathway analysis.

**Additional file 4.** Length distribution of Protein-Coding Region prediction from BLAST.

**Additional file 5.** Length distribution of Protein-Coding Region prediction from EST scan.

**Additional file 6.** The analysis of RNA-seq data identified 50 CP genes and 20 predicted CP genes in alate adults (ARs), WRs (workers) and SRs (soldiers).

**Additional file 7.** The five selected genes and their primers used in qRT-PCR analysis.

## Data Availability

All data analyzed during this study are included in this article and its additional files. All raw sequence reads have been deposited in the NCBI SRA database and are accessible through SRA accession number SRP199695. The assembled gene sequences have been deposited in the NCBI TSA database under accession number GHMS00000000.

## Abbreviations

BmorCPH24: Name of endocuticle gene
cDNA: Complementary de oxy ribonucleic acid
CDS: Protein coding regions
CPs: Structural cuticular proteins
DEG: Differentially expressed genes
dNTPs: Deoxy ribonucleic acid triphosphate
FDR: False discovery rate
GO: Gene ontology
KEGG: Kyoto encyclopedia of gene and genome
KOG: Eukaryotic orthologues group of genes
mRNA: Messenger ribonucleic acid
qRT-PCR: Quantitative real time polymerase chain reaction
RNAi: Ribonucleic acid interference
RPKM: Reads per kilo base per million
RR-1 and RR-2: Two groups of cuticular protein CPR family
SgAbd-1-like, SgAbd-2, SgAbd-9, Abd-5, SgAbd-2-like and Abd-4-like: Names of endocuticle genes
SOAP: Short oligonucleotide analysis package
