# Supplementary material for: Comparative transcriptomic analysis and endocuticular protein gene expression of alate adults, workers and soldiers of the termite Reticulitermes aculabialis
Source: BMC Genomics. 2019 Oct 15;20:742. doi: 10.1186/s12864-019-6149-4 (PMC6794787; doi:10.1186/s12864-019-6149-4)
Supplement: Supplementary file 1 — Additional file 1. Unigenes length distribution of the R. aculabialis unigenes. [file 12864_2019_6149_MOESM1_ESM.pdf]

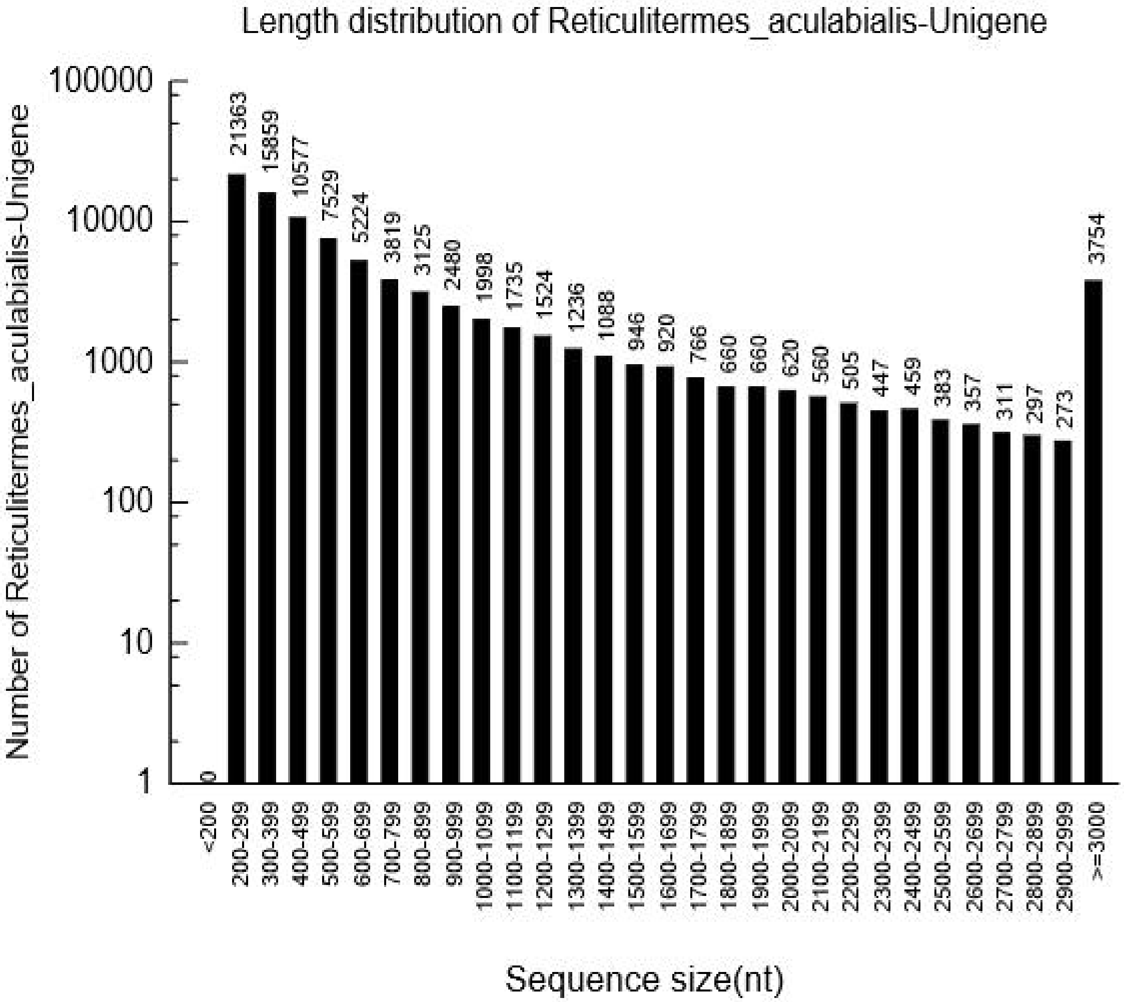

Additional file 1 Distribution of the lengths of the *R. aculabialis* unigenes. The histogram presents the sequence length distribution for the identified significant matches. The x-axis indicates the sequence size from 200 nt to >300 nt. The y-axis indicates the number of unigenes for every given size. The results of the sequence length matches (with a cut-off E-value of 1.0E-5) the Nr database were greater among the longer assembled sequences.
