# Supplementary material for: Comparative transcriptomic analysis and endocuticular protein gene expression of alate adults, workers and soldiers of the termite Reticulitermes aculabialis
Source: BMC Genomics. 2019 Oct 15;20:742. doi: 10.1186/s12864-019-6149-4 (PMC6794787; doi:10.1186/s12864-019-6149-4)
Supplement: Supplementary file 2 — Additional file 2. Functional annotation of the R. aculabialis transcriptome. [file 12864_2019_6149_MOESM2_ESM.pdf]

Additional file 2 Functional annotation of the *R. aculabialis* transcriptome. We used BLASTX to query various protein databases and to annotate 89,475 unigene sequences. All of the unigenes were annotated against the Nr, Swiss-Prot, COG and KEGG databases.

Total Unigenes	Nr	Swissprot	KEGG	COG	Annotation genes	Without Annotation genes
89,475	26,556	15,886	10,605	14,256	26,645	62,830
