# Supplementary material for: Comparative transcriptomic analysis and endocuticular protein gene expression of alate adults, workers and soldiers of the termite Reticulitermes aculabialis
Source: BMC Genomics. 2019 Oct 15;20:742. doi: 10.1186/s12864-019-6149-4 (PMC6794787; doi:10.1186/s12864-019-6149-4)
Supplement: Supplementary file 3 — Additional file 3. DEGs enrichment by the KEGG pathway analysis. [file 12864_2019_6149_MOESM3_ESM.pdf]

Additional file 3 DEGs enrichment by the KEGG pathway analysis. (a) In comparing alates and soldiers, only 1 pathway was significantly changed. (b)In comparing alates and workers, 25 pathways were significantly changed.(c)Between workers and soldiers, 19 pathways were significantly changed (Q-value <0.05).

a	Pathway	Q-value
1	Starch and sucrose metabolism	0.006459

b	Pathway	QVALUE
1	Ribosome	0.000000
2	Glycine, serine and threonine metabolism	0.000052
3	Phagosome	0.000060
4	Citrate cycle (TCA cycle)	0.000860
5	MAPK signaling pathway	0.003181
6	Histidine metabolism	0.003181
7	Estrogen signaling pathway	0.006165
8	Antigen processing and presentation	0.006165
9	Starch and sucrose metabolism	0.006223
10	Insect hormone biosynthesis	0.006223
11	Drug metabolism - cytochrome P450	0.006648
12	Focal adhesion	0.008045
13	Pyruvate metabolism	0.008143

14	Glycolysis / Gluconeogenesis	0.009896
15	Regulation of actin cytoskeleton	0.012333
16	Adherens junction	0.027445
17	Dopaminergic synapse	0.027445
18	Thyroid hormone signaling pathway	0.027445
19	Oxytocin signaling pathway	0.027445
20	Proximal tubule bicarbonate reclamation	0.027445
21	Retinol metabolism	0.027445
22	Pentose and glucuronate interconversions	0.027445
23	Phenylalanine metabolism	0.045751
24	Carbon metabolism	0.045820
25	Tryptophan metabolism	0.045820

c	Pathway	QVALUE
1	Ribosome	0.000000
2	Phagosome	0.000000
3	Focal adhesion	0.000019
4	Regulation of actin cytoskeleton	0.000145
5	Adherens junction	0.000387
6	Gap junction	0.000505
7	Glycolysis / Gluconeogenesis	0.001962
8	Estrogen signaling pathway	0.007660
9	Antigen processing and presentation	0.007660
10	Carbon metabolism	0.010535
11	Citrate cycle (TCA cycle)	0.013565
12	Pyruvate metabolism	0.039930
13	Biosynthesis of amino acids	0.039930
14	MAPK signaling pathway	0.039930

15	Longevity regulating pathway - worm	0.039930
16	Oxytocin signaling pathway	0.039930
17	Proximal tubule bicarbonate reclamation	0.039930
18	Glycine, serine and threonine metabolism	0.041970
19	Drug metabolism - cytochrome P450	0.044892
