# Supplementary material for: Comparative transcriptomic analysis and endocuticular protein gene expression of alate adults, workers and soldiers of the termite Reticulitermes aculabialis
Source: BMC Genomics. 2019 Oct 15;20:742. doi: 10.1186/s12864-019-6149-4 (PMC6794787; doi:10.1186/s12864-019-6149-4)
Supplement: Supplementary file 4 — Additional file 4. Length distribution of Protein-Coding Region prediction from BLAST. [file 12864_2019_6149_MOESM4_ESM.pdf]

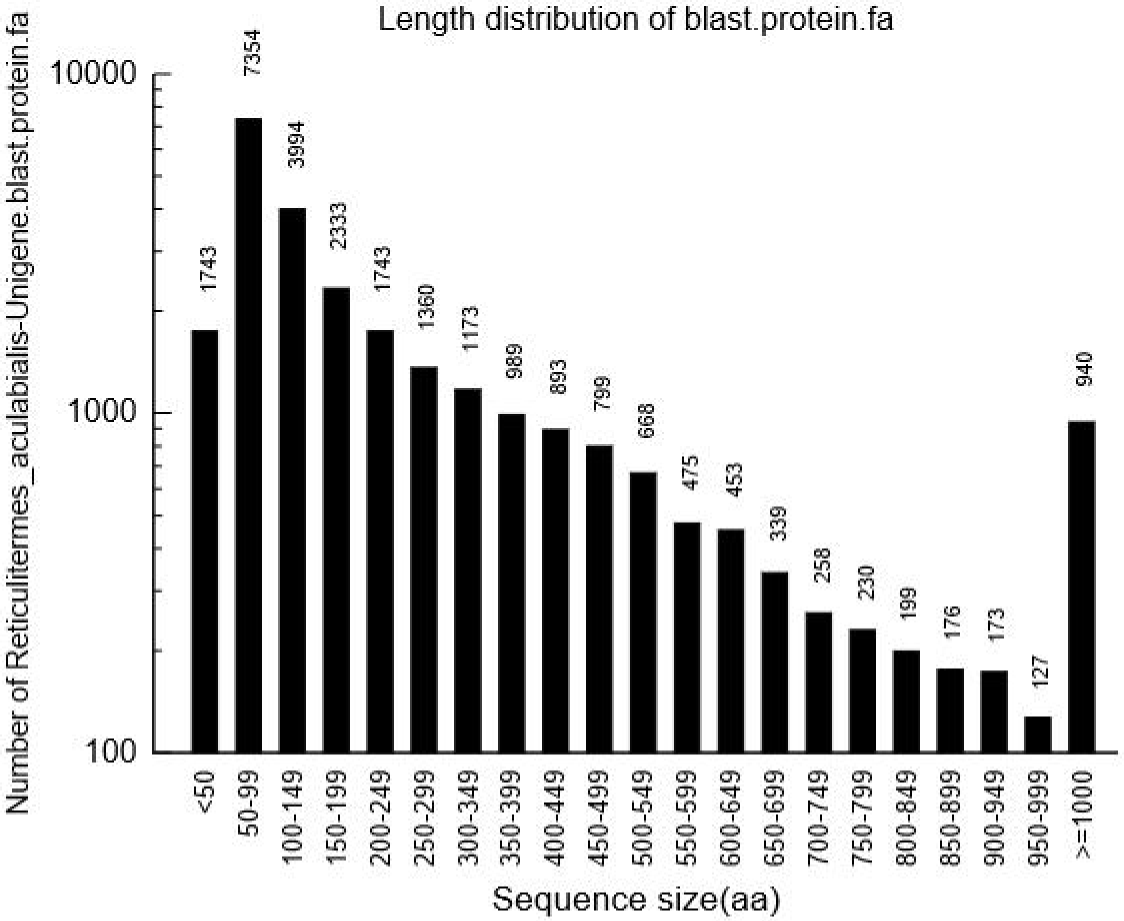

Additional file 4 Length distribution of Protein-Coding Region prediction from BLAST. 26,419 unigenes were predicted using BLASTX. Histogram presentation of sequence-length distribution for significant matches that was found. The x-axis indicates sequence size from 0 nt to >1000aa. The y-axis indicates the number of unigenes for every given size.
