# Supplementary material for: Comparative transcriptomic analysis and endocuticular protein gene expression of alate adults, workers and soldiers of the termite Reticulitermes aculabialis
Source: BMC Genomics. 2019 Oct 15;20:742. doi: 10.1186/s12864-019-6149-4 (PMC6794787; doi:10.1186/s12864-019-6149-4)
Supplement: Supplementary file 6 — Additional file 6. The analysis of RNA-seq data identified 50 CP genes and 20 predicted CP genes in alate adults (ARs), WRs (workers) and SRs (soldiers). [file 12864_2019_6149_MOESM6_ESM.pdf]

Additional file 6 The analysis of RNA-seq data identified 50 CP genes and 20 predicted CP genes in alate adults (ARs), WRs (workers) and SRs (soldiers). The plus sign (+) indicated that the CP genes were detected in alate adults, worker and soldiers. The minus sign (-) indicated that the CP genes were not detected in ARs, WRs and SRs.

Unigene ID	Annotation	ARs	WRs	SRs
Unigene0001094	Endocuticle structural glycoprotein SgAbd-9, partial [*Zootermopsis nevadensis*]	+	+	+
Unigene0001350	Endocuticle structural glycoprotein SgAbd-2, partial [*Zootermopsis nevadensis*]	+	+	+
Unigene0003968	Endocuticle structural glycoprotein ABD-5 [*Zootermopsis nevadensis*]	+	+	+
Unigene0006451	Endocuticle structural glycoprotein SgAbd-2, partial [*Zootermopsis nevadensis*]	+	+	+
Unigene0016014	Endocuticle structural glycoprotein SgAbd-9, partial [*Zootermopsis nevadensis*]	+	-	+
Unigene0034952	Endocuticle structural glycoprotein SgAbd-2 [*Zootermopsis nevadensis*]	+	+	+
Unigene0005776	Larval cuticle protein A3A [Zootermopsis nevadensis]	+	+	+
Unigene0001622	Cuticle protein 5 [*Zootermopsis nevadensis*]	+	+	+
Unigene0006737	Cuticle protein 6 [*Zootermopsis nevadensis*]	+	+	+
Unigene0007006	Pro-resilin [*Zootermopsis nevadensis*]	+	+	-
Unigene0007427	Cuticle protein 7 [*Zootermopsis nevadensis*]	+	+	+
Unigene0007781	Cuticle protein 8 [*Zootermopsis nevadensis*]	+	+	+
Unigene0007782	Cuticle protein 8 [*Zootermopsis nevadensis*]	+	+	+
Unigene0012271	Cuticle protein 19.8 [*Zootermopsis nevadensis*]	-	-	-
Unigene0015864	Cuticle protein 19 [*Zootermopsis nevadensis*]	-	+	-
Unigene0015865	Cuticle protein 19 [*Zootermopsis nevadensis*]	-	-	-
Unigene0003615	Larval cuticle protein A3A [*Zootermopsis nevadensis*]	+	+	+
Unigene0016689	Cuticle protein 19 [*Zootermopsis nevadensis*]	-	-	-
Unigene0016690	Cuticle protein 19 [*Zootermopsis nevadensis*]	-	-	-
Unigene0018725	Cuticle protein 5 [*Zootermopsis nevadensis*]	+	+	+
Unigene0020452	Insect cuticle protein, partial [*Oryctes borbonicus*]	+	+	+

Unigene0022565	Cuticle protein 19 [*Zootermopsis nevadensis*]	+	+	-
Unigene0022937	Larval cuticle protein 2 [*Zootermopsis nevadensis*]	+	+	+
Unigene0023251	Cuticle protein [Zootermopsis nevadensis]	-	+	+
Unigene0026602	Cuticle protein 19 [Zootermopsis nevadensis]	+	+	+
Unigene0027695	Cuticle protein 2 [Zootermopsis nevadensis]	+	+	+
Unigene0030512	Cuticle protein [Zootermopsis nevadensis]	+	+	+
Unigene0030513	Cuticle protein [Zootermopsis nevadensis]	+	+	+
Unigene0031921	Cuticle protein 19 [Zootermopsis nevadensis]	+	+	+
Unigene0032805	ecdysone inducible protein 75 isoform A [*Blatella germanica*]	+	+	+
Unigene0000360	Cuticle protein 5 [*Zootermopsis nevadensis*]	+	+	+
Unigene0036100	pupal cuticle protein 78E [*Culex quinquefasciatus*]	+	+	+
Unigene0036958	Naked cuticle-like protein [*Zootermopsis nevadensis*]	+	+	+
Unigene0036959	Naked cuticle-like protein [*Zootermopsis nevadensis*]	+	+	+
Unigene0041964	Cuticle protein 7 [*Zootermopsis nevadensis*]	+	+	+
Unigene0043703	RecName: Full=Cuticle protein 1; AltName: Full=Bc-NCP1	+	+	+
Unigene0044478	Larval cuticle protein A3A [*Zootermopsis nevadensis*]	+	+	+
Unigene0044479	Cuticle protein [*Zootermopsis nevadensis*]	-	+	+
Unigene0044480	Larval cuticle protein A3A [*Zootermopsis nevadensis*]	+	+	+
Unigene0044727	Cuticle protein 2 [*Zootermopsis nevadensis*]	+	+	+
Unigene0049904	Cuticle protein [*Daphnia magna*]	+	+	+
Unigene0054816	dumpy, isoform X [*Drosophila melanogaster*]	+	+	+
Unigene0055158	Cuticle protein 6 [*Zootermopsis nevadensis*]	+	+	+
Unigene0060365	Cuticle protein 19 [*Zootermopsis nevadensis*]	+	-	-
Unigene0060731	Larval cuticle protein A2B [*Zootermopsis nevadensis*]	+	+	+
Unigene0062249	Cuticle protein 19 [*Zootermopsis nevadensis*]	-	+	-
Unigene0068946	Cuticle protein 19 [*Zootermopsis nevadensis*]	+	+	+
Unigene0077231	Cuticle protein 19 [*Zootermopsis nevadensis*]	+	+	+
Unigene0081694	Cuticle protein 6 [*Zootermopsis nevadensis*]	+	+	-

Unigene0081860	Cuticle protein 5 [*Zootermopsis nevadensis*]	+	+	+
Unigene0003047	PREDICTED: pupal cuticle protein 20-like isoform X2 [*Nicrophorus vespilloides*]	+	+	+
Unigene0004905	PREDICTED: cuticle protein 12.5-like [*Panthera Tigris altaica*]	+	+	+
Unigene0007914	PREDICTED: cuticle protein 16.5-like [*Dendroctonus ponderosae*]	+	+	+
Unigene0010997	PREDICTED: cuticle protein 19-like [*Apis florea*]	-	-	+
Unigene0018405	PREDICTED: larval cuticle protein A2B-like [*Papilio polytes*]	-	+	-
Unigene0019248	PREDICTED: endocuticle structural glycoprotein SgAbd-2-like [Cimex lectularius]	+	+	+
Unigene0020343	PREDICTED: adult cuticle protein 1-like [*Anoplophora glabripennis*]	+	+	+
Unigene0022979	PREDICTED: cuticle protein 7 [*Bactrocera oleae*]	+	+	-
Unigene0033089	PREDICTED: cuticle protein 19 [*Cimex lectularius*]	+	+	+
Unigene0034144	PREDICTED: cuticle protein 70, isoforms A and B-like [*Halyomorpha halys*]	+	+	+
Unigene0035856	PREDICTED: endocuticle structural glycoprotein ABD-4-like [*Cimex lectularius*]	+	+	+
Unigene0036615	PREDICTED: cuticle protein 19.8-like [*Diuraphis noxia*]	+	+	+
Unigene0040223	PREDICTED: endocuticle structural glycoprotein SgAbd-1-like [*Bemisia tabaci*]	-	+	+
Unigene0052246	PREDICTED: cuticle protein 16.5-like [*Monodelphis domestica*]	+	+	+
Unigene0058974	PREDICTED: larval cuticle protein A2B-like [*Bemisia tabaci*]	+	+	+
Unigene0060894	PREDICTED: cuticle protein 7-like [*Microplitis demolitor*]	-	-	-
Unigene0069784	PREDICTED: endocuticle structural glycoprotein SgAbd-2-like isoform X2 [*Agrilus planipennis*]	+	+	+
Unigene0071342	PREDICTED: cuticle protein CP14.6 [*Tribolium castaneum*]	+	+	+
Unigene0078792	PREDICTED: larval cuticle protein A2B-like [*Copidosoma floridanum*]	-	+	+

Unigene0085584	PREDICTED: cuticle protein 16.8-like [*Rhagoletis zephyria*]	+	+	+
