# Supplementary material for: Comparative transcriptomic analysis and endocuticular protein gene expression of alate adults, workers and soldiers of the termite Reticulitermes aculabialis
Source: BMC Genomics. 2019 Oct 15;20:742. doi: 10.1186/s12864-019-6149-4 (PMC6794787; doi:10.1186/s12864-019-6149-4)
Supplement: Supplementary file 7 — Additional file 7. The five selected genes and their primers used in qRT-PCR analysis. [file 12864_2019_6149_MOESM7_ESM.pdf]

Additional file 7 The five selected genes and their primers used in qRT-PCR analysis

Unigene ID	Annotation	Primer sequences
	Beta-actin (reference gene)	Forward: AGCGGGAAATCGTGCGTGAC<br>Reverse: CAATGGTGATGACCTGGCCAT
Unigene0003968	Endocuticle structural glycoprotein ABD-5 [*Zootermopsis nevadensis*]	Forward: AATTCCAGACAAGTGACGGCATATCC<br>Reverse: CTCCAAGGCTTCGTTGTCGGTTC
Unigene0016014	Endocuticle structural glycoprotein SgAbd-9, partial [*Zootermopsis nevadensis*]	Forward:GACATCGTTGTTACTTACATCGCTGAC<br>Reverse: CTTCGCTGAGTCTCGCTTCTTCC
Unigene0019248	Endocuticle structural glycoprotein SgAbd-2-like [*Cimex lectularius*]	Forward: GCAGAAGGACTTGGAAGCACAGG<br>Reverse: GCCATTCTCGTCAGCCGTGTATG
Unigene0034952	Endocuticle structural glycoprotein SgAbd-2 [*Zootermopsis nevadensis*]	Forward: GCTCCTTCTCCTACATTGCACCAG<br>Reverse: GGTGGAGGCGTAGGCAGGTG
Unigene0035856	Endocuticle structural glycoprotein ABD-4-like [*Cimex lectularius*]	Forward: ACTCGGATCGTCCTCACCTACAC<br>Reverse: CATCGCCTGCCTCGTTCTCTTC
